# Identification of the Chemical Constituents in Aqueous Extract of Zhi-Qiao and Evaluation of Its Antidepressant Effect

**DOI:** 10.3390/molecules20046925

**Published:** 2015-04-16

**Authors:** Ming Wu, Hongwu Zhang, Chao Zhou, Hongmei Jia, Zhuo Ma, Zhongmei Zou

**Affiliations:** 1 Department of Food and Pharmaceutical, Hubei University of Technology, Wuhan 430068, China; E-Mail: hbwuming@163.com; 2 Institute of Medicinal Plant Development, Chinese Academy of Medical Sciences and Peking Union Medical College, Beijing 100193, China; E-Mails: 18101318775@163.com (H.Z.); smilezhouchao@163.com (C.Z.); jia090909@126.com (H.J.)

**Keywords:** *Citrus aurantium*, UPLC-Q-TOF/MS, antidepressant, PC12 cells

## Abstract

The immature fruit of *Citrus aurantium* L. (Zhi-Qiao, ZQ) has been used as a traditional medicine in China. Our previous study has shown that ZQ decoction may contribute to the antidepressant-like action of Chaihu-Shu-Gan-San. However, there are no reports on the chemical constituents of ZQ aqueous extract or its anti-depression effects. Firstly, this research reported the on-line identification of the chemical constituents in the aqueous extract of ZQ by coupling ultra-performance liquid chromatography/time-of-flight mass spectrometry (UPLC-Q-TOF/MS). A total of 31 chemical constituents were identified in ZQ aqueous extract, including one tannic acid, five flavones, 13 flavanones, one limonoid, three coumarins, three cyclic peptides, and five polymethoxylated flavonoids. The antidepressant effect of ZQ aqueous extract was evaluated *in vivo* and the results indicated that the mice immobility time during the forced swimming test and the tail suspension test were significantly reduced with ZQ treatment. MTT assays showed both ZQ aqueous extract and its major constituents (naringin, hesperidin, neohesperidin, and nobiletin) had neuroprotective effect on corticosterone-induced neurotoxicity in PC12 cells. The *in vivo* and *in vitro* results suggest that ZQ has an antidepressant effect.

## 1. Introduction

*Citrus aurantium* L. (bitter orange) has been widely cultivated in tropics and subtropics. The extract of its immature fruit or peel is added to many weight loss formulas in dietary supplements. The immature fruit of *C. aurantium* (Zhi-Qiao, ZQ) has also been used as a traditional medicine in China for treating the stagnation of dyspepsia, improving gastrointestinal function, and reducing chest pain [1,2].

The chemical composition of ZQ includes flavonoids (naringin, hesperidin, neohesperidin) [3], alkaloids (synephrine) [4], and coumarins (meranzin, auraptene) [5]. With the development of the LC-MS instrument, it has become more popular for direct identification of multiple components in a complicated matrix [6]. Ultra-performance liquid chromatography/time-of-flight mass spectrometry [7] and atmospheric pressure chemical ionization mass spectrometry [8] have been applied in characterization of polymethoxylated flavonoids (PMFs) in the ethyl acetate extract of ZQ. Unfortunately, these works lack of identification of accurate structures. Recently, Chen *et al.* [9] investigated the antioxidants in the methanol extract of ZQ and identified 25 flavones with a HPLC-DAD-MS method.

Pharmacological studies have revealed that ZQ has diverse bioactivities, including anti-tumor [10], anti-oxidation [11], anti-virus [12], anti-inflammation [13], anti-bacterial [14], anti-allergic [15], and effects on mammalian metabolism [16]. The alcohol extract of ZQ could improve the sucrose preference test and reduce the forced swimming time on the chronic unpredicted mild stress (CUMS) model of depression in rats [17]. ZQ is one of the key herbs in Chaihu-Shu-Gan-San (CSGS), a traditional Chinese medicines (TCMs) formula for treatment of depression clinically in China [18]. During our research on antidepressant effect of CSGS, we found that CSGS lost some of the important regulatory action on the disturbance of metabolic pathways related to depression, when one of the herbs ZQ was removed from CSGS [19]. These findings encouraged us to explore the contributions of ZQ to the antidepressant-like action of CSGS.

Traditionally, the most commonly used form of TCMs is a decoction. However, there are no reports on the chemical constituents of ZQ decoction or its anti-depression effect. Consequently, in this paper the on-line identification of the multiple components in the aqueous extract of ZQ was performed using an efficient and sensitive UPLC-Q-TOF/MS method. Then, the antidepressant effects of ZQ aqueous extract were evaluated using a forced swimming test and tail suspension test. Further, the neuroprotective effects of ZQ aqueous extract and its main chemical constituents (naringin, hesperidin, neohesperidin, and nobiletin) were determined on corticosterone-induced neurotoxicity in PC12 cells.

## 2. Results and Discussion

### 2.1. Optimization of LC and MS Conditions

In order to cover the overall constituents of ZQ aqueous extract with good resolution in a short analysis, UPLC parameters, including column temperature (30 °C, 35 °C, and 40 °C), mobile phases systems (methanol-aqueous, acetonitrile-aqueous, methanol-aqueous with 0.1% formic acid, and acetonitrile-aqueous with 0.1% formic acid), and gradient program were examined. Subsequently, the MS conditions including the desolvation gas flow (600, 800, and 1,200 L/H), capillary voltage (2.5, 3.0, and 3.5 kV in positive ion mode; 2.0, 2.5, and 3.0 kV in negative ion mode), and the cone voltage (30, 35, and 40 V) were also optimized. The total peak area was taken as criteria for optimization. As a result, the optimum conditions were determined as described in Section 3.3.

### 2.2. Identification of Chemical Constituents in ZQ Aqueous Extract

**Figure 1:**
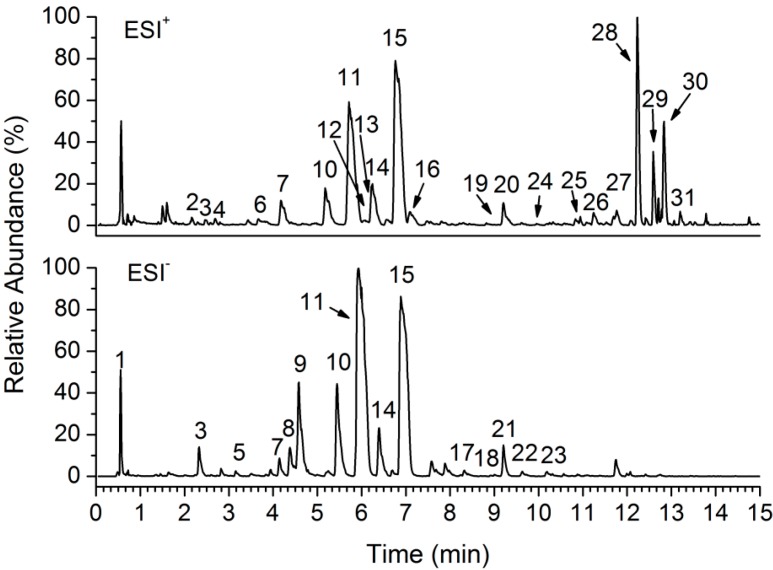
Typical UPLC-Q-TOF/MS base peak intensity (BPI) chromatograms of ZQ aqueous extract in positive and negative ion modes.

**Figure 2:**
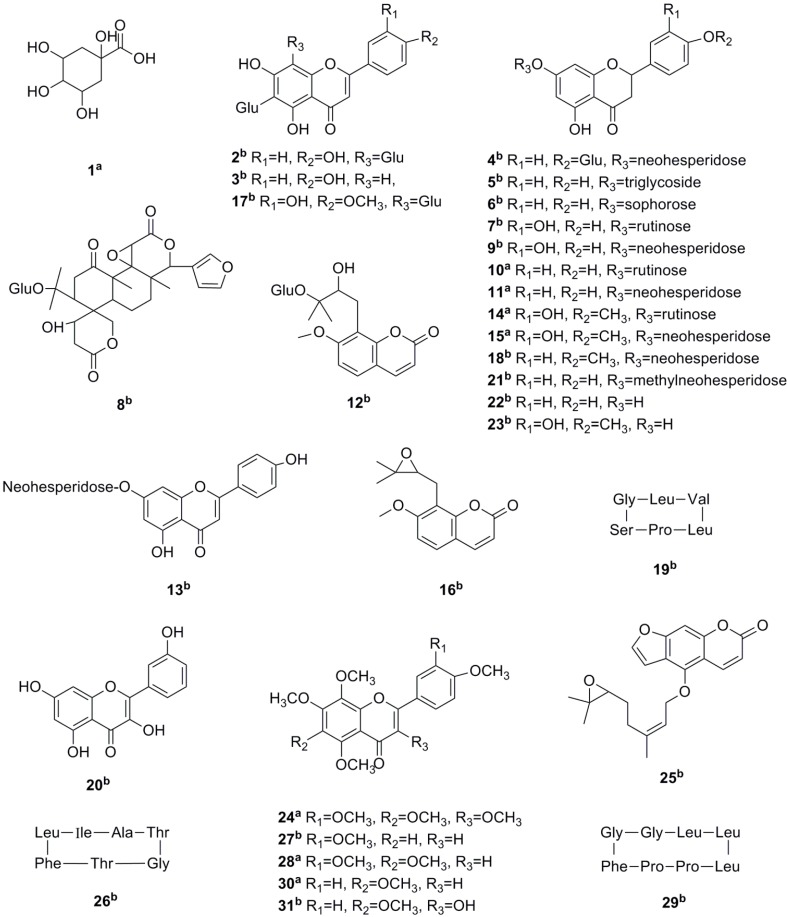
The chemical structures of compounds identified in ZQ aqueous extract; ^a^ Structurally confirmed by comparison with reference chemicals; ^b^ Structure assignment tentative, based on MS and literature data.

The reference substances and ZQ sample were analyzed by using the optimized UPLC-Q-TOF/MS method. The base peak intensity (BPI) chromatograms of ZQ sample in positive and negative ESI modes are shown in Figure 1. For most of the constituents, [M−H]^−^, [M+H]^+^ and/or [M+Na]^+^ adduct ions were observed. Due to the use of formic acid in mobile phase, the [M+46−H]^−^ fragment ions corresponding to [M+HCOOH−H]^−^ were also observed in negative ion mode. The results provided valuable information for confirming accurate molecular weights and the composition of the constituents. Based on the retention behaviors, accurate molecular weight and MS^n^ fragment data, 31 compounds from the ZQ aqueous extract were tentatively identified (Figure 2) by comparison with reference substances and literature data (Table 1). The identified compounds can be classified into seven classes including one tannic acid (peak 1), five flavones (peaks 2, 3, 13, 17, 20), 13 flavanones (peaks 4, 5, 6, 7, 9, 10, 11, 14, 15, 18, 21, 22, 23), one limonoid (peak 8), three coumarins (peak 12, 16, 25), three cyclic peptides (peak 19, 26, 29), and five polymethoxylated flavonoids (peaks 24, 27, 28, 30, 31). 

**Table 1:** Identification of the chemical constituents in ZQ aqueous extract by UPLC-Q-TOF/MS analysis.

Peak No.	Identification	Rt ^c^ (min)	Formula	Positive Ion Mode of ESI-MS (*m*/*z*)		Negative Ion Mode of ESI-MS (*m*/*z*)	
				Quasi-molecular ion	MS^2^ ions	Quasi-molecular ion	MS^2^ ions
1	Quinic acid ^a^	0.55	C_7_H_12_O_6_	-	-	191.0558 [M−H]^−^	165.0363
2	6,8-Di-glucopyranocylapigenin ^b^	2.16	C_27_H_30_O_15_	595.1670 [M+H]^+^	577.1560; 457.1149	-	-
3	Isovitexin ^b^	2.39	C_21_H_20_O_10_	433.1233 [M+H]^+^	379.0893; 367.0936; 313.1040	431.0986 [M−H]^−^	353.1286
4	Glucosyl-naringin ^b^	2.68	C_33_H_42_O_19_	765.2200 [M+Na]^+^	625.1794; 581.1855; 539.1867	-	-
5	Naringenin-7-*O*-triglycoside ^b^	3.15	C_33_H_42_O_19_	-	-	741.2241 [M−H]^−^	433.1134
6	Naringenin-7-*O*-sophorose ^b^	3.71	C_27_H_32_O_15_	597.1827 [M+H]^+^	435.1441; 417.1241; 199.1124	-	-
7	Eriocitrin ^b^	4.18	C_27_H_32_O_15_	597.1796 [M+H]^+^	289.0714; 179.0316; 163.0404	595.1669 [M−H]^−^	287.0620
8	Ichangin-4-*O*-β-d-glucopyranoside ^b^	4.38	C_32_H_42_O_14_	-	-	649.2505 [M−H]^−^	605.2636; 443.2061
9	Neoeriocitrin ^b^	4.59	C_27_H_32_O_15_	-	-	595.1658 [M−H]^−^	459.1152
10	Narirutin ^a^	5.19	C_27_H_32_O_14_	603.1685 [M+Na]^+^	581.1850; 503.1530; 435.1188; 273.0711	579.1719 [M−H]^−^	271.0622
11	Naringin ^a^	5.71	C_27_H_32_O_14_	603.1693 [M+Na]^+^	581.1850; 503.1530; 435.1188; 273.0711	579.1724 [M−H]^−^	459.1138; 271.0649
12	Meranzin-*O*-glucoside ^b^	5.84	C_21_H_28_O_10_	463.1578 [M+Na]^+^	419.1343	-	-
13	Rhoifolin ^b^	5.87	C_27_H_30_O_14_	579.1698 [M+H]^+^	503.1538; 355.1575; 273.0760	-	-
14	Hesperidin ^a^	6.25	C_28_H_34_O_15_	633.1792 [M+Na]^+^	449.1434; 413.1336; 303.0869	609.1833 [M−H]^−^	301.0723; 286.0500; 151.0063
15	Neohesperidin ^a^	6.81	C_28_H_34_O_15_	633.1794 [M+Na]^+^	449.1434; 413.1336; 303.0873	609.1816 [M−H]^−^	489.1423; 343.0804; 301.0660
16	Meranzin ^b^	7.09	C_15_H_16_O_4_	261.1139 [M+H]^+^	189.0554	-	-
17	6,8-Di-glucopyranocyldiosmetin ^b^	8.25	C_28_H_32_O_16_	-	-	623.1945 [M−H]^−^	503.1146
18	Neoponcirin ^b^	8.94	C_28_H_34_O_14_	-	-	593.1899 [M−H]^−^	285.0778
19	Cyclo(-Gly-Leu-Val-Leu-Pro-Ser-) ^b^	9.20	C_27_H_46_N_6_O_7_	589.3329 [M+Na]^+^	567.3504; 454.2654	-	-
20	Kaempferol ^b^	9.25	C_15_H_10_O_6_	287.0924 [M+H]^+^	239.2353	-	-
21	Fumotonaringin ^b^	9.29	C_28_H_34_O_14_	-	-	593.1853 [M−H]^−^	285.0767
22	Naringenin ^b^	9.63	C_15_H_12_O_5_	-	-	271.0613 [M−H]^−^	151.0041
23	Hesperitin ^b^	10.19	C_16_H_14_O_6_	-	-	301.0760 [M−H]^−^	286.0501; 242.0572
24	3-Methoxynobiletin ^a^	10.24	C_22_H_24_O_9_	433.1486 [M+H]^+^	403.1021; 373.0571	-	-
25	Epoxybergamottin ^b^	10.85	C_21_H_22_O_5_	355.1518 [M+H]^+^	344.0939	-	-
26	Citrusin I ^b^	10.94	C_34_H_53_N_7_O_9_	726.3782 [M+Na]^+^	704.3984 591.3105	-	-
27	Isosinensetin ^b^	11.56	C_20_H_20_O_7_	373.1264 [M+H]^+^	358.1040; 343.1270	-	-
28	Nobiletin ^a^	12.23	C_21_H_22_O_8_	403.1397 [M+H]^+^	383.1766; 239.1505	-	-
29	Cyclo(-Gly-Gly-Leu-Leu-Leu-Pro-Pro-Phe-) ^b^	12.78	C_41_H_62_N_8_O_8_	817.4586 [M+Na]^+^	795.4759; 682.3929; 399.2105	-	-
30	Tangeretin ^a^	12.82	C_20_H_20_O_7_	373.1288 [M+H]^+^	358.1063; 343.0814	-	-
31	7-Hydroxyl-4',3,5,6,8-pentamethoxy-flavone ^b^	13.20	C_20_H_20_O_8_	389.1240 [M+H]^+^	374.1106; 359.0764; 197.0739	-	-

^a^ Structurally confirmed by comparison with reference chemicals; ^b^ Structure assignment tentative, based on MS and literature data; ^c^ Rt: retention time.

**Figure 3:**
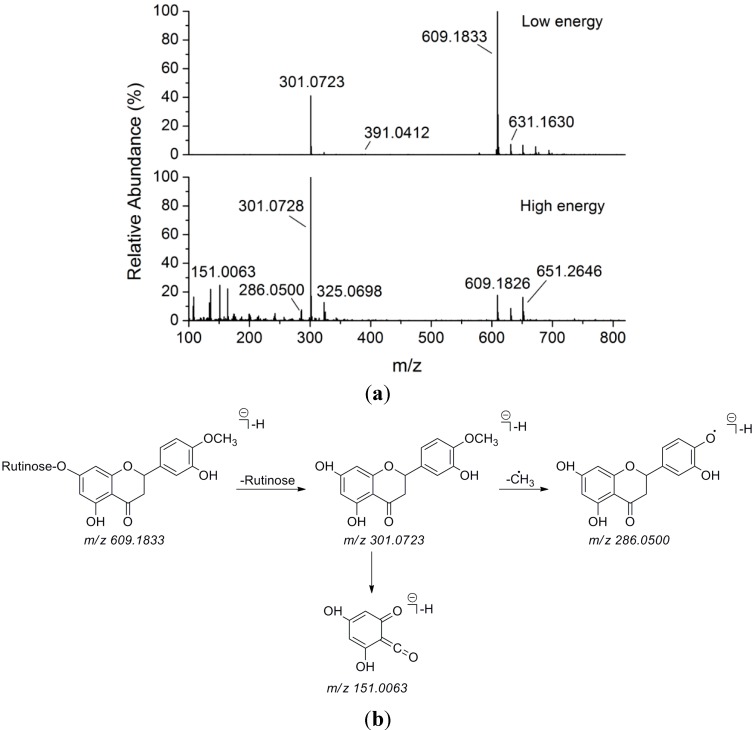
(**a**) ESI(−)-MS and MS^E^ spectra of hesperidin (**14**); (**b**) ESI-MS/MS fragmentation pattern of hesperidin (**14**).

Here, an ion at the retention time of 6.25 min (peak 14) is taken as an example to illustrate the identification process. The base peak of its [M−H]^−^ at *m*/*z* 609.1833, as well as its [M+H]^+^ at *m*/*z* 633.1792, is indicative of the molecular formula as C_28_H_34_O_15_. Additionally, the neutral loss of 308 Da (C_12_H_20_O_9_) from C_28_H_33_O_15_^−^ in the MS^E^ spectra was attributed to the characteristic ion [M−H−rutinose]^−^ fragments. Then, the loss of 15 Da indicated the existence of “–CH_3_” group. The fragment ion at *m*/*z* 151.0063 (C_7_H_3_O_4_^−^) was produced from flavanone nucleus. Thus, this compound was tentatively identified as hesperidin, and further confirmed by comparing with reference compound [20]. Spectra of ion fragments in MS^E^ analysis and the proposed fragmentation pattern of hesperidin in negative ion mode were shown in Figure 3.

Three cyclic peptides (compounds **19**, **26**, and **29**) were tentatively identified by analysis of their ESI(+)-MS spectra (Figures S1–S3 in the Supplementary Material) and compared with literature data [21,22]. For example, compound **19** had [M+Na]^+^ at *m*/*z* 589.3329, as well as [M+H]^+^ at *m*/*z* 567.3504, is indicative of the molecular formula as C_27_H_46_N_6_O_7_. These data suggested that compound **19** was a cyclic peptide consisting of six amino acids. The ions at *m*/*z* 454.2654, 355.1535, 242.1149, and 185.0933 suggested the sequence of Leu, Val, Leu, and Gly. However, we did not find evidence for the connection of the other amino acids (Ser, Pro) in MS/MS spectrum. Thus, we tentatively identified compound **19** as cyclo(-Gly-Leu-Val-Leu-Pro-Ser-), which has been previously reported from *Citrus aurantium* [23]. Similiarly, compounds **26** and **29** were tentatively identified as citrusin I, and cyclo(-Gly-Gly-Leu-Leu-Leu-Pro-Pro-Phe-), respectively.

### 2.3. Effect of ZQ Aqueous Extract on Immobility Time in FST and TST

**Figure 4:**
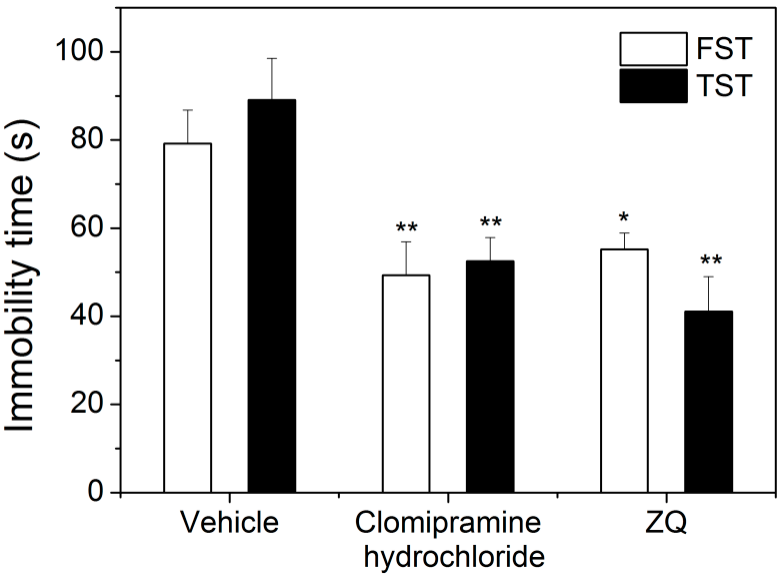
Effect of ZQ on FST and TST in mice (mean ± SD) (*n* = 10). *p < 0.05, **p < 0.01 as compared with vehicle.

The forced swimming test (FST) and tail suspension test (TST) are the most widely used animal models for antidepressant activity screening, partially because of their high predictive validity [24]. In the present study, oral administration of ZQ aqueous extract produced a marked reduction on immobility time in the FST (*p* < 0.05), similar to the positive control clomipramine hydrochloride. Furthermore, the significantly decrease on immobility time in the TST (*p* < 0.01) was also observed after treatment with the aqueous extract of ZQ. Both of the FST and TST experiments are suggestive of ZQ aqueous extract with significant antidepressant-like effect (Table 2 and Figure 4).

**Table 2:** Effect of ZQ on FST and TST in mice (mean ± SD) (*n* = 10).

Groups	FST		TST	
	Immobility Time (s)	Shorten Rate (%)	Immobility Time (s)	Shorten Rate (%)
Vehicle	79.2 ± 7.6		89.1 ± 9.4	
Clomipramine hydrochloride	49.3 ± 7.6 **	37.8	52.5 ± 5.4 **	41.1
ZQ	55.2 ± 3.7 *	33.3	41.1 ± 7.9 **	53.9

* *p* < 0.05, ** *p* < 0.01 as compared with control.

### 2.4. Neuroprotective Effect of ZQ Aqueous Extract on Corticosterone-Induced Neurotoxicity in PC12 Cells

**Figure 5:**
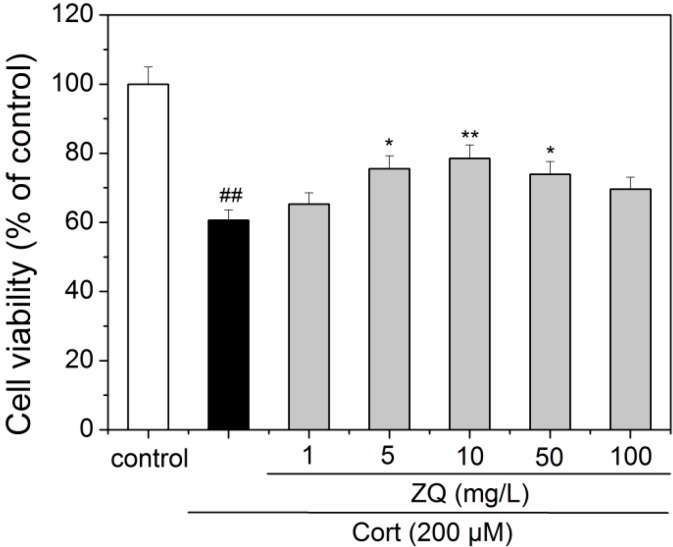
Effect of ZQ on the cell viability in corticosterone-treated PC12 cells. The results are expressed as mean ± SD (*n* = 3). **^##^** *p* < 0.01 as compared with control group; * *p* < 0.05 and ** *p* < 0.01 as compared with the corticosterone group. ZQ: ZQ aqueous extract; Cort: corticosterone.

The PC12 cell line, derived from rat pheochromocytoma tumors, possesses typical neuron features and expresses a high level of glucocorticoid receptors. The PC12 cells treated with high concentration of glucocorticoid to induce the neuronal damage have been widely used as an *in vitro* experimental model of depression [25,26]. PC12 cells were treated with 200 μM of corticosterone in the absence or presence of ZQ aqueous extract in varying concentrations for 48 h. Then, cell viability was measured by MTT assay. The results showed that treatment with 200 μM of corticosterone could induce cytotoxicity in PC12 cells. However, different concentrations of ZQ aqueous extract (1, 5, 10, 50, and 100 mg/L) significantly increased the cell viability, and the survival rates were 65.3%, 75.5%, 78.6%, 73.9%, and 69.6% of control, respectively (Figure 5).

### 2.5. Neuroprotective Effect of Four Major Components in ZQ Aqueous Extract on Corticosterone-Induced Neurotoxicity in PC12 Cells

**Figure 6:**
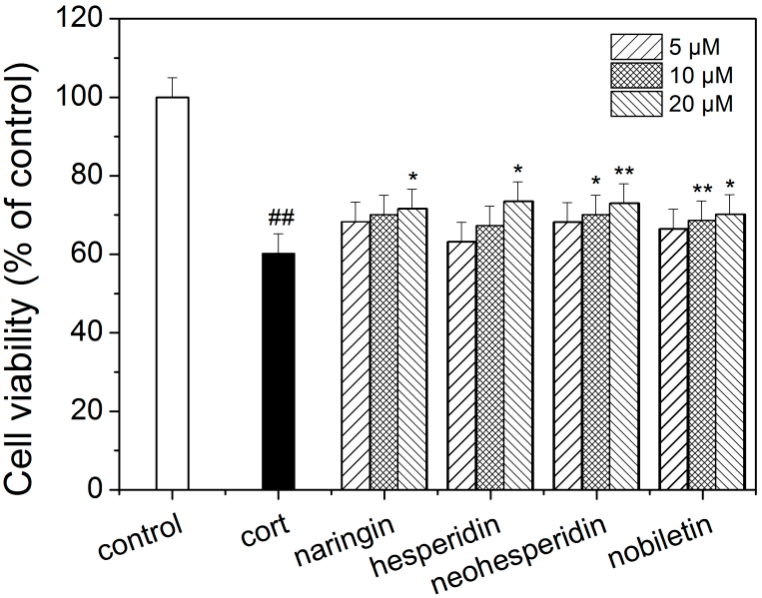
Effect of major components on the cell viability in corticosterone-treated PC12 cells. The results are expressed as mean ± SD (*n* = 3). **^##^** *p* < 0.01 as compared with control group; * *p*< 0.05 and ** *p*< 0.01 as compared with the corticosterone group. Cort: corticosterone.

The protection of four major components from ZQ aqueous extract against corticosterone-induced neurotoxicity in PC12 cells was also evaluated. When the cells were treated with naringin, hesperidin, neohesperidin, and nobiletin at 5, 10, 20 μM in the presence of 200 μM of corticosterone for 48 h, the cell viability was significantly increased as compared with the corticosterone treated group (Figure 6).

### 2.6. Discussion

In present study, we identified 31 constituents in the ZQ extracts using UPLC-Q-TOF/MS and the antidepressant effect of ZQ aqueous extract had antidepressive-like effect in both the FST and TST assays in mice. In order to explore its mechanism of action, the neuroprotective effect of both ZQ aqueous extract and its four major constituents was evaluated on corticosterone-induced neurotoxicity in PC12 cells. Naringin, hesperidin, neohesperidin, and nobiletin had protection against corticosterone-induced neurotoxicity in PC12 cells in a dose-dependent manner. ZQ aqueous extract also showed protection at concentrations of 5 and 10 mg/L, but had cytotoxicity at high concentrations (50, 100 mg/L). The results indicated that neuroprotective effect of ZQ may be involved in antidepressant mechanism. Its major active constituents should be naringin, hesperidin, neohesperidin, and nobiletin. Obviously, there exist constituents with cytotoxicity on PC12 cells in ZQ aqueous extract. 

Flavonoids are a large group of phenolic compounds that are widely distributed in plants, and are known to antidepressant-like activity at least in animal models of depression [27]. Many of flavonoids, such as rutin, quercetin and hesperidin, have a significant antidepressant effect in the FST [28]. It has been reported that the potential mechanism of hesperidin could be involved in modulation of the l-arginine-NO-cGMP pathway [29]. Nobiletin also has antidepressant-like effect in both the FST and TST in mice [30]. Our previous study reports that naringin, hesperidin, and neohesperidin show inhibition against monoamine oxidase (MAO) [19]. Thus, the antidepressant-like effect and the mechanism of action of those flavonoids from ZQ aqueous extract require further exploration.

## 3. Experimental Section

### 3.1. Chemicals

Reference substances of quinic acid, narirutin, naringin, hesperidin, neohesperidin, 3-methoxynobiletin, nobiletin, and tangeretin were purchased from the National Institute for the Control of Pharmaceutical and Biological Products (Beijing, China). All of the purities were above 98% by HPLC analysis. Clomipramine hydrochloride tablets were purchased from Jiangsu En Pharmaceutical Co. Ltd. (Xuzhou, China). Dulbecco’s Modified Eagle Medium (DMEM), fetal bovine serum, heat-inactivated horse serum, penicillin and streptomycin were purchased from Gibco (Grand Island, NY, USA). Corticosterone and MTT were purchased from Sigma-Aldrich (St. Louis, MO, USA). HPLC grade acetonitrile and methanol were purchased from Fisher (Waltham, MA, USA). Formic acid (HPLC grade) was purchased from Tedia (Fairfield, OH, USA). Water was prepared using a Millipore Milli-Q purification system (Bedford, MA, USA).

### 3.2. Plant Material and Sample Preparation

The raw herb was purchased from Beijing Tongren Tang Pharmaceutical Co. Ltd. (Beijing, China) and identified as the immature fruits of *Citrus aurantium* L. by Yulin Lin of the Institute of Medicinal Plant Development (IMPLAD), Chinese Academy of Medical Sciences and Peking Union Medical College. The voucher specimen is deposited in our laboratory of IMPLAD. 

The raw herb was soaked in distilled water (1:10, *w*/*v*) for 0.5 h at room temperature and thereafter boiled for 1 h. The filtrate was collected and the residue was then boiled again for 1 h. The filtrates were combined, concentrated under vacuum and lyophilized to give extract. The yield of ZQ extract was 20.28%. The accurately weighed ZQ extract (0.5 g) was dissolved in 25 mL of 50% methanol (*v*/*v*), and centrifuged at 13,000 rpm for 15 min at 4 °C. The 2 μL was injected for UPLC-Q-TOF/MS analysis after filtration through a 0.22 μm membrane filter. All samples were analyzed in triplicate.

### 3.3. UPLC-Q-TOF/MS System

The sample was analyzed on a Waters Acquity^TM^ Ultra Performance LC system (Waters Corporation, Milford, MA, USA) equipped with an Acquity UPLC HSS T3 column (100 mm × 2.1 mm, 1.7 μm, Waters Corporation) at a column temperature of 35 °C. The mobile phase was composed of water (A) and acetonitrile (B), each containing 0.1% formic acid. The line gradient program was carries out as follows: 10%–14% B at 0–2 min; 14%–20% B at 2–6 min; 20%–30% B at 6–9 min; 30%–40% B at 9–11 min; 40%–70% B at 11–14 min; 70%–99% B at 14–16 min; 99% B at 16–18 min; and 10% B at 18–20 min. The flow rate was 0.45 mL/min. The mass spectrometric data were collected using a Q-TOF analyzer in a SYNAPT HDMS system (Waters Corporation) in both positive and negative ion modes. The parameters were set as previously described [31]. The source temperature was set at 120 °C with a cone gas flow of 50 L/H, a desolvation gas temperature of 450 °C and a desolvation gas flow of 800 L/H. For the positive and negative ion modes, the capillary voltage was set to 3.0 kV and 2.5 kV, respectively, and the cone voltage was set to 35 V. Centroid data were collected from *m*/*z* 50 to 1200 with a scan time of 0.3 s and an interscan delay of 0.02 s over a 15 min analysis time. Leucine-enkephalin was used as the lock mass (*m*/*z* 556.2771 in positive mode and *m*/*z* 554.2615 in negative mode) at a concentration of 0.5 μg/mL with a flow rate of 80 μL/min. The lock spray frequency was set at 20 s.

### 3.4. Animals and Treatments

Thirty male ICR mice, weighing 18–22 g were purchased from the Institute of Laboratory Animal Science, CAMS and PUMC (Beijing, China). The mice were housed individually in cages and maintained (23 ± 2 °C and 40%–60% humidity) under a standard 12-h light/dark cycle with free access to purified water and commercial diet. The mice were habituated for 7 days before the experiment. All experimental procedures were approved by the Ethics Committee of the Institute of Medicinal Plant Development, CAMS & PUMC. The mice were randomly divided into three groups. The mice in the vehicle, positive control and ZQ treated groups were administrated with normal saline (0.9% NaCl), clomipramine hydrochloride (40 mg/kg) and ZQ (equivalent to 6 g crude drug/kg body weight), for 14 consecutive days, respectively.

### 3.5. Forced Swimming Test Assay

The forced swimming test was performed according to the conventional method of Porsolt [32]. Briefly, thirty minutes after the last drug administration, each mouse was forced to swim for 6 min in a glass cylinder (20 cm × 14 cm) containing fresh water up to a height of 12 cm at 25 ± 1 °C. The duration of immobility was recorded for the last 4 min by two independent observers blinded to the treatments. All FSTs were recorded using a video camera.

### 3.6. Tail Suspension Test Assay

The tail suspension test was performed as previously described [33]. Briefly, thirty minutes after the last drug administration, mice were individually suspended by its tail using a clamp (2 cm from the end) for 6 min in a box (25 × 25 × 30 cm) with the head 5 cm from the bottom. Testing was carried out in a darkened room with minimal background noise. The duration of immobility was recorded for the last 4 min by two independent observers blinded to the treatments. All TSTs were recorded using a video camera.

### 3.7. Cell Culture and Treatment

PC12 cells were obtained from the American Type Culture Collection (Rockville, MD, USA). PC12 cells were maintained in DMEM medium supplemented with penicillin (100 U/mL), streptomycin (100 μg/mL), 5% fetal bovine serum and 10% horse serum at 37 °C in humidified atmosphere of 95% air and 5% CO_2_.

The appropriate damaging concentration of corticosterone was selected based on the results of Gao [34]. In brief, different concentrations of corticosterone (10, 50, 100, 200, and 400 μM) were incubated with PC12 cells for 48 h, and the cell viability was determined by MTT. When treated with 200 μM corticosterone for 48 h, the cell viability decreased to approximately 60%, which induced cell injury without inducing cell death, and was used in subsequent experiments.

To research the neuroprotective effect of ZQ and its major components, the experimental design contained the treatment groups as follows: non-treated control, 200 μM of corticosterone, 200 μM of corticosterone plus ZQ (1, 5, 10, 50, and 100 mg/L), and 200 μM of corticosterone plus naringin, hesperidin, neohesperidin, and nobiletin (5, 10, 20 μM). In the experiments, PC12 cells were seeded on a 96-well culture plates for 24 h, then corticosterone was added 48 h prior to treatment with ZQ or its major components, and then the cells were co-incubated with corticosterone and ZQ or its major components for another 24 h.

### 3.8. Cell Viability Assay

Cell survival was evaluated by 3-(4,5-dimethylthiazol-2-yl)-2,5-diphenyltetrazolium bromide (MTT) assay. Briefly, PC12 cells were seeded on a 96-well culture plates at a density of 1 × 10^5^ cells/well. At the end of the treatment, the media was removed. Then the cells were washed with D-Hanks, and MTT solution (final concentration, 0.5 mg/mL) was added and further incubated for 4 h at 37 °C. Subsequently, the dark blue formazan crystals formed in intact cells were solubilized with DMSO. After shaking at room temperature for 10 min, absorbance at 570 nm was measured with a microplate reader (Bio-Rad 550, Hercules, CA, USA). Cell viability was expressed as a percentage of the non-treated control.

### 3.9. Statistical Analysis

The results were presented as mean ± standard deviation (SD). Data were analyzed using one-way analysis of variance (ANOVA) followed by post-hoc LSD test and differences were considered statistically significant at p < 0.05. All experiments were performed in triplicate.

## 4. Conclusions

In the present study, an efficient and sensitive method employing ultra-performance liquid chromatography/time-of-flight mass spectrometry (UPLC-Q-TOF/MS) was developed for qualitative analysis of chemical constituents of ZQ aqueous extract. A total of 31 compounds including one tannic acid, five flavones, 13 flavanones, one limonoid, three coumarins, three cyclic peptides, and five polymethoxylated flavonoids were identified. The results from FST and TST indicated that ZQ aqueous extract owned antidepressant effect. Our results also showed that the protections of ZQ aqueous extract against corticosterone-induced neurotoxicity in PC12 cells. Four major components (naringin, hesperidin, neohesperidin, and nobiletin) displayed the protection against corticosterone-induced cytotoxicity in PC12 cells in a dose-dependent manner, a further clue they are main chemical constituents of ZQ responsible for its antidepressant effect [19]. 

## Supplementary Materials

Supplementary materials can be accessed at: http://www.mdpi.com/1420-3049/20/04/6925/s1.
