# Supplementary material for: Identification of the Chemical Constituents in Aqueous Extract of Zhi-Qiao and Evaluation of Its Antidepressant Effect
Source: Molecules. 2015 Apr 16;20(4):6925–40. doi: 10.3390/molecules20046925 (PMC6272419; doi:10.3390/molecules20046925)
Supplement: Supplementary file 1 [file molecules-20-06925-s001.pdf]

Supplementary Materials

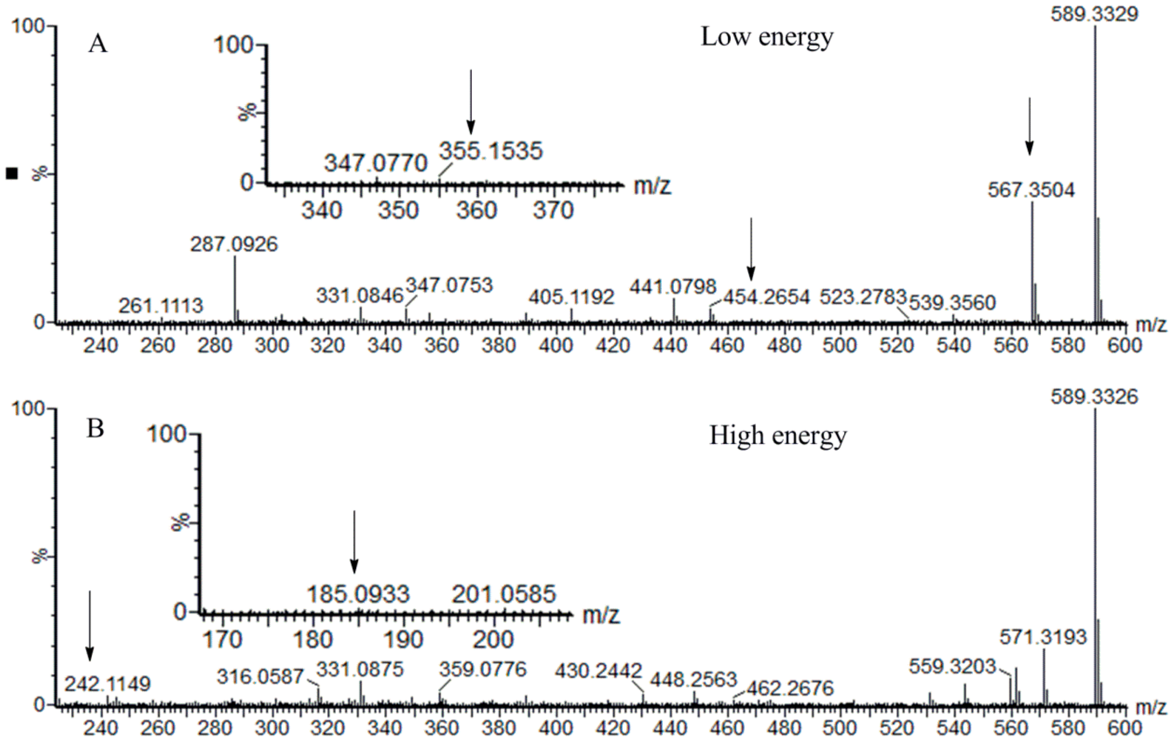

Figure S1. The low energy (**A**) and high energy (**B**) MS^E spectra of ion at *m*/*z* 589.3329 (**19**).

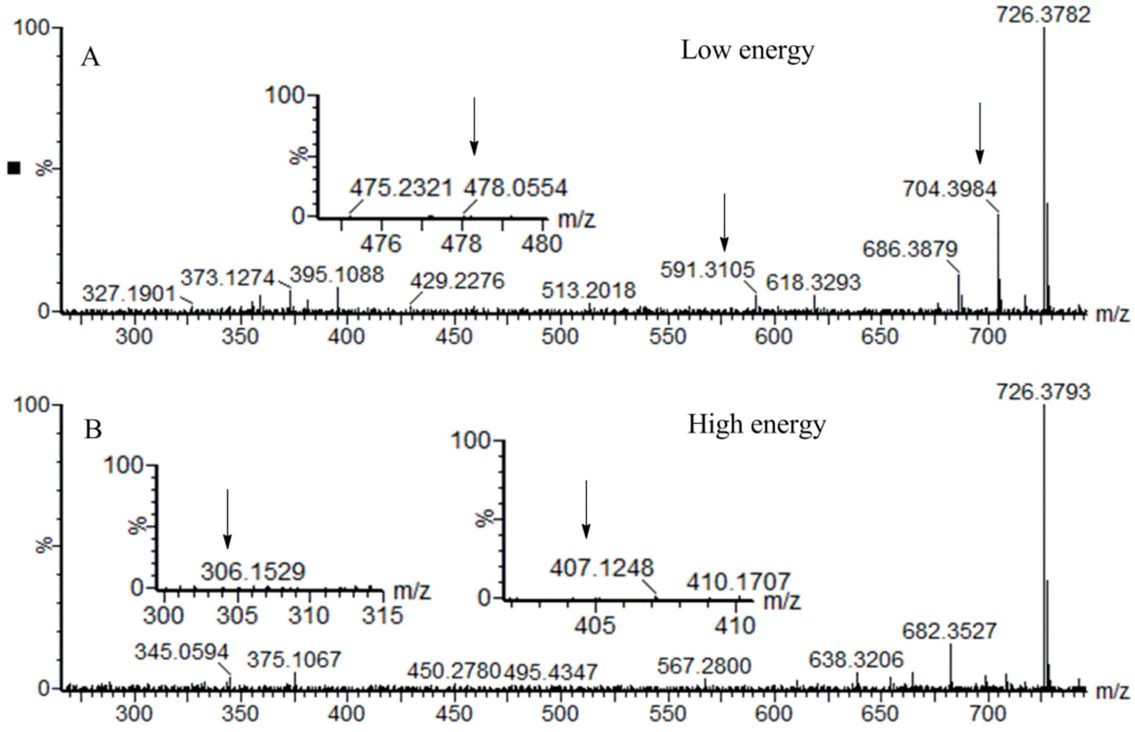

Figure S2. The low energy (**A**) and high energy (**B**) MS^E spectra of ion at *m*/*z* 726.3782 (**26**).

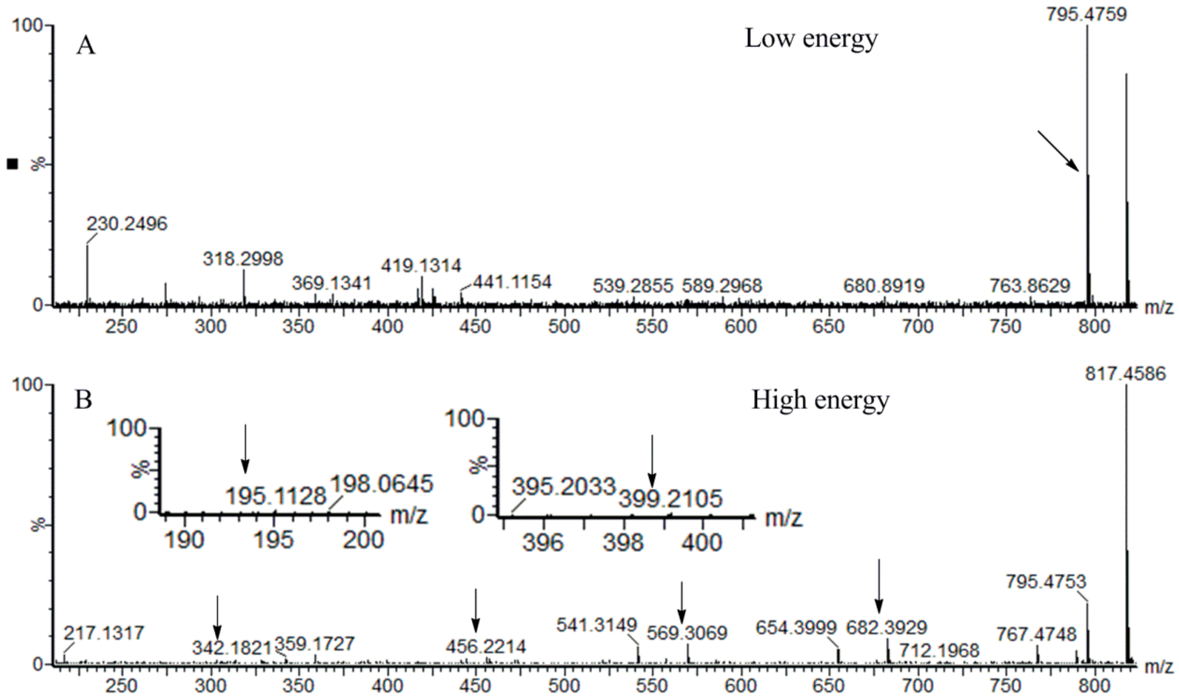

Figure S3. The low energy (**A**) and high energy (**B**) MS^E spectra of ion at *m/z* 817.4586 (**29**).
